# Role of noncoding RNA in drug resistance of prostate cancer

**DOI:** 10.1038/s41419-021-03854-x

**Published:** 2021-06-08

**Authors:** Lifeng Ding, Ruyue Wang, Danyang Shen, Sheng Cheng, Huan Wang, Zeyi Lu, Qiming Zheng, Liya Wang, Liqun Xia, Gonghui Li

**Affiliations:** Department of Urology, Sir Run Run Shaw Hospital, Zhejiang University School of Medicine, Hangzhou, China

**Keywords:** Prostate cancer, Prostate cancer

## Abstract

Prostate cancer is one of the most prevalent forms of cancer around the world. Androgen-deprivation treatment and chemotherapy are the curative approaches used to suppress prostate cancer progression. However, drug resistance is extensively and hard to overcome even though remarkable progress has been made in recent decades. Noncoding RNAs, such as miRNAs, lncRNAs, and circRNAs, are a group of cellular RNAs which participate in various cellular processes and diseases. Recently, accumulating evidence has highlighted the vital role of non-coding RNA in the development of drug resistance in prostate cancer. In this review, we summarize the important roles of these three classes of noncoding RNA in drug resistance and the potential therapeutic applications in this disease.

## Facts

- Androgen-deprivation treatment and chemotherapy are indispensable treatments for metastatic prostate cancer (PCa). However, drug resistance is hard to avoid.
- Anti-tumor drugs cause a change in the expression of noncoding RNA, thus affecting the drug sensitivity of PCa.
- Noncoding RNAs are proposed as candidate biomarkers to predict the drug response of PCa.
- Noncoding RNAs are proposed as a potential therapeutic target to reverse drug resistance of PCa.

## Open questions

- How do noncoding RNAs mediate drug resistance in PCa?
- How can noncoding RNAs be used as biomarkers to predict the drug response of PCa?
- How can noncoding RNAs be used to design drug targets and reverse the drug resistance of PCa?

## Introduction

Prostate cancer is the most commonly diagnosed malignancy in men worldwide^1^. It is particularly prevalent in the West, while the incidence is lower in Eastern Asian^2^. Apart from race, lifestyle factors such as smoking, body mass index, and physical activity also contribute to prostate cancer^3^. Because of the coverage of screening and early detection, there are more than 1.2 million newly diagnosed prostate cancer patients annually and more than 350,000 deaths worldwide^4^. Androgen deprivation treatment (ADT) is the initial treatment used for prostate cancer^5^. Moreover, it is reported that androgen deprivation treatment combined with chemotherapy drugs can improve the survival of prostate cancer^6^. However, as with many drugs, a large proportion of patients who do benefit from initial chemotherapy become resistant to chemotherapy drugs^7^. Hence, it is urgent to uncover the detailed molecular mechanism of drug resistance in prostate cancer, and thus find a way to maximize the benefits of chemotherapy.

Early research on carcinogenesis focused mainly on protein-coding genes, because proteins are considered central to molecular biology^8^. However, many noncoding RNAs species have been discovered due to the development of transcriptional sequencing^9^. In addition, it has been verified that numerous noncoding RNAs participate in many vital cellular functions and in disease, especially in cancer^10^. According to their size, noncoding RNAs can be divided into two groups: (1) small noncoding RNAs (sncRNAs), with length less than 200 nucleotides(nt), including microRNAs and piRNAs, (2) long noncoding RNA (lncRNAs), including circRNAs and pseudogenes^10^.

**Fig. 1:**
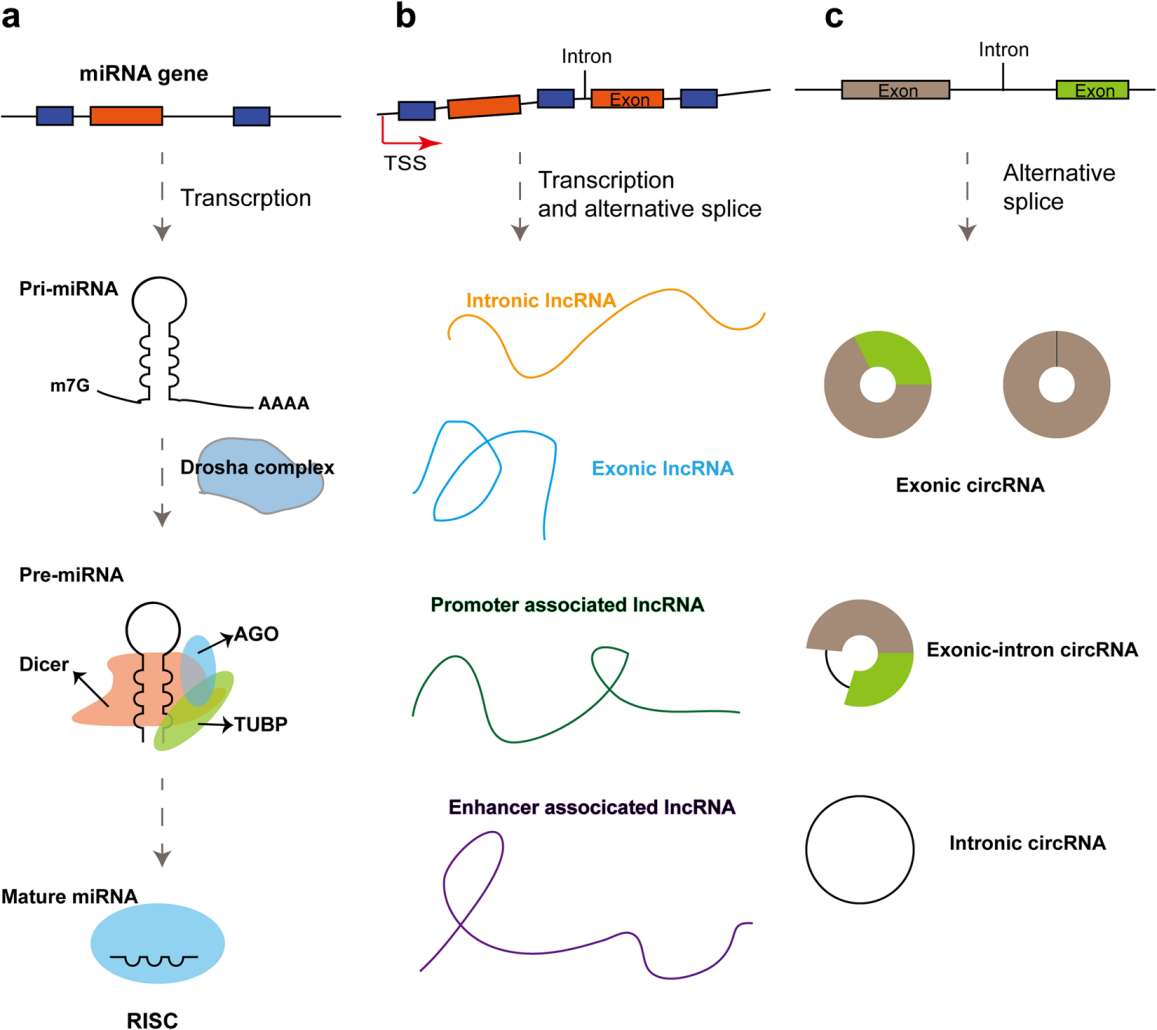
Biogenesis of several noncoding RNAs. **a** Transcription of miRNAs is regulated by RNA polymerase II. The pri-miRNAs are processed by several consecutive cleavages to produce mature miRNAs since the pri-miRNAs are transcripted. Finally, mature miRNAs are incorporated into the Argonaute to form miRNA-induced silencing complex (RISC). **b** According to the different origin transcription sites, lncRNAs can be divided into various types: intronic lncRNAs, exonic lncRNAs, promoter-associated lncRNAs, and enhancer-associated lncRNAs. **c** Most circRNAs are derived from the pre-mRNA. Due to the different compositions, circRNAs are classified into several types, including exonic circRNAs, exonic-intronic circRNAs, and intronic circRNAs.

In this review, we discuss the characteristics and vital role of noncoding RNAs, especially miRNA, lncRNA, and circRNA, in drug resistance of prostate cancer. These noncoding RNAs are potential therapeutic targets for treating drug resistance in prostate cancer^5,11^ (Fig. 1).

## Evidence acquisition

We accessed PubMed to search English-language articles up to October 2020, using a combination of the following terms: noncoding RNA, or microRNA, or miRNA, or lncRNA, or long noncoding RNA, or circular RNA, or circRNA, and prostate cancer, and drug resistance or chemoresistance.

## MicroRNA and drug resistance

MiRNA is a type of conserved small noncoding RNA whose length is about 18–22 nucleotides. Mature miRNA can directly target the 3’ untranslated region (UTR) of mRNA, as some target to the 5’ UTR or to the coding sequence, in a sequence-specific manner. As a result, miRNA can downregulate the expression level of mRNAs by hampering the translational process or mRNA decay^11,12^. Thus, miRNA has been shown to take part in carcinogenesis by regulating the expression level of important oncogenes or tumor suppressor genes^13–15^. miRNAs also play a vital role in drug resistance. Here, we present some crucial miRNAs involved in drug resistance of prostate cancer.

### miRNA and resistance to anti-androgen drugs

Indeed, androgen-deprivation treatment was one of the earliest hormonal therapies in oncology^16,17^. Since the discovery of the androgen receptor (AR), ADT has been an indispensable treatment for prostate cancer. The first generation of antiandrogen drugs, Cyproterone (CPA), is a type of steroidal drugs, which competitively binds to the AR^18^. However, the inevitable adverse effects, such as loss of libido and impotence, restrict the clinical use of CPA. The presence of next-generation anti-androgen drug flutamide, which is a non-steroidal anti-androgen drug, largely avoids those adverse effects^19^. In addition, emerging anti-androgen drugs like enzalutamide, which specifically bind to the LBD of the AR, show highly promising effects in patients with castration-resistant prostate cancer. However, despite the rapid development of anti-androgen drugs, there is still a large proportion of prostate cancer patients that becomes resistant^20^. Therefore, it is meaningful to uncover the underlying mechanism of resistance to anti-androgen drugs.

Growing evidence elucidates that miRNAs have a vital role in anti-androgen drugs resistance (Table 1). Pimenta et al. reported that miR-23b and miR-27b can sensitize castration prostate cancer cells to flutamide by targeting CCNG1^21^. Another group revealed that miR-221 and miR-222, which are significantly up-regulated in CRPC cells, can maintain the castration resistance phenotype in prostate cancer^22^. In addition, miR-663 is involved in the prostate cancer castration phenotype. miR-663 significantly alters the effect of the AR signal but does not alter the expression of the AR to induce castration-therapy resistance. Evaluation of 117 prostate patients’ specimens also confirmed that miR-663 was upregulated in CRPC patients and could be a prognostic indicator for clinical recurrence^23^.

**Table 1:** MiRNA and castration resistance in prostate cancer.

MiRNAs	Expression	Genes and pathways	Reference
miR-23b and miR-27b	Down	CCNG1	^21^
miR-221 and miR-222	Up	P21/Kip1	^22^
miR-212	Down	hnRNPH1/AR, AR-V7	^24^
miR-150–5p and miR-150–3p	Down	SPOCK1	^98^
miR-616	Up	TFPI-2	^99^
miR-663	Up	KCNC4, DHRS7, NKX3.1, DHCR24, PSMA7	^23^
miR-32	Up	BTG2	^100^
miR-361–3p	Down	AR-v7 and MKNK2	^25^
miR-4719 and miR-6756–5p	Up	IL-24	^101^
miR-4638–5p	Down	Kidins220	^102^
miR-100–5p	Up	MTOR	^103^

Meanwhile, some miRNAs can also target AR and androgen splicing variant 7 (AR-V7) to re-sensitize drug resistant prostate cancer cells^24,25^. miR-212 was found downregulated in prostate cancer tissues compared to adjacent tissues. Moreover, overexpression of miR-212 can restrain the castration resistance of prostate cancer by inhibiting hnRNPH1 and in turn reducing the expression of the AR and AR-v7^24^. Expect from miR-212, miR-361–3p was also reported to increase the enzalutamide sensitivity of prostate cancer via targeting the AR-v7. MiR-361–3p can directly bind to the 3’ UTR of AR-v7 and MKNK2 in hypoxia conditions to sensitize prostate cancer cells to enzalutamide^25^.

Apart from those miRNAs which have a role in the castration phenotype, miRNAs also could be promising prognostic biomarkers in castration-resistant prostate cancer (Table 2). Huang et.al. analyzed miRNA expression in two independent cohorts, including a screening cohort which contained 23 patients and a follow-up cohort with 100 patients. They found that high expression levels of miR-1290 and miR-375 were associated with a poor survival rate. What’s more, miR-1290 and miR-375 also have good performance in prediction of CRPC stage^26^.

**Table 2:** Clinical application of miRNA in prostate cancer.

MiRNAs	Expression	Potential clinical application	Reference
miR-1290 and miR-375	Up	Prognostic markers	^26^
miR-216a	Up	Prognostic markers	^104^

### miRNA and resistance to taxane

Taxane, composed of paclitaxel and docetaxel, is a class of well-known anti-tumor drugs which affect the intrinsic instability of microtubules^27,28^. Paclitaxel (PXL) was the first taxane to become clinically available. It can arrest the cell cycle by binding to the tubulin, which in turn stabilizes the microtubule structure^29^. Docetaxel (DXL) is the first-line drug to treatment metastatic castration-resistant prostate cancer (mCRPC) and provides a significant advantage in CRPC survival^30^. However, taxane therapy inevitably encounters the problem of resistance, despite a good response to initial treatment^31^. Hence, a deeper understanding of the underlying mechanism of taxane resistance would provide opportunities to overcome taxane resistance and dramatically improve patient survival.

Several miRNAs have been reported as upregulated in three paclitaxel-resistant cell lines, including miR-200b-3p, miR-375, and miR-34b-3p. In addition, the downstream genes of miRNAs, LARP1, and CCND1 are increased in paclitaxel-resistant cell lines, which could be the potential cause of resistance^32^. It has been found that miR199a, which is downregulated in prostate cancer tissues, is suppressed in PTX-resistance cell lines. MiR-199a can reverse the paclitaxel resistance by suppressing the expression of YES1^33^. Similarly, miR-148a attenuates paclitaxel resistance by reducing the expression of MSK1^34^. MiR-34a, another miRNA which was found downregulated in paclitaxel-resistant cells, increases the chemosensitivity by directly targeting JAG1/Notch1 axis^35^ (Table 3).

**Table 3:** MiRNA and chemoresistance in prostate cancer.

MiRNAs	Expression	Genes and pathways	Drug	Reference
miR-148a	Down	MSK1	Paclitaxel	^34^
miR-199a	Down	YES1	Paclitaxel	^33^
miR-34a	Down	JAG1/Notch1	Paclitaxel	^35^
miR-375	Up	SEC23A/YAP1	Docetaxel	^36^
miR-323	Up	P73	Docetaxel	^37^
miR-181a	Up	ABCB1	Docetaxel	^7^
miR-195	Down	CLU	Docetaxel	^39^
miR-27a	Up	P53	Docetaxel	^105^
miR-204	Down	ZEB1	Docetaxel	^40^
miR-143	Down	KRAS	Docetaxel	^41^
miR-193a-5p	Up	Bach2	Docetaxel	^38^
miR-138	Up	Kindlin-2	Docetaxel	^106^
miR-200b	Down	Bmi-1	Docetaxel	^42^
miR-205	Down	RAB27A/LAMP3	Cisplatin	^47^
miR-17-92 cluster	Up	AKT pathway	Cisplatin	^48^

In addition to the research on paclitaxel, there are other studies focused on docetaxel, which is considered as the first-line treatment for mCRPC. Wang et al. found that expression of miR-375 was elevated after docetaxel treatment, and in vivo and in vitro assays also confirmed that miR-375 could increase docetaxel resistance by targeting SEC23A and YAP1^36^. Similarly, miR-323 was identified as having a high expression level in docetaxel-resistant cells by other groups. miR-323 significantly increased the inhibitory concentration (IC50) value for docetaxel in prostate cancer cell lines by repressing the expression level of P73^37^. In addition, miR-193–5p was found to enhance drug resistance to docetaxel in prostate cancer by inhibiting Bach2 expression^38^. Aside from elevated expression of miRNAs in docetaxel-resistant prostate cancer, there are several miRNAs which can reverse docetaxel resistance. MiR-195 was reported to be downregulated in the DOC-resistant prostate cancer cells compared to the DOC-sensitive prostate cancer cells. In addition, high expression of miR-195 lowers the IC50 of DOC through decreasing the expression level of CLU^39^. miR-204 can also directly inhibit the expression of ZEB1 and then attenuate docetaxel resistance^40^. miR-143, a well-known miRNA which has been comprehensively studied in various types of cancer, has been proved to play a vital role in docetaxel resistance of prostate cancer. Xu et al. revealed that miR-143 could target the EGFR/RAS/MAPK pathway to enhance the docetaxel sensitivity of prostate cancer cells, and this is a potential site for the treatment of docetaxel-resistant prostate cancer^41^. In another study analyzing 30 prostate cancer patients’ specimens, miR-200b was identified as a tumor-suppressor gene and promoter of chemosensitivity in prostate cancer^42^.

### miRNA and resistance to cisplatin

Since prostate cancer often becomes refractory to hormonal treatment and taxane drugs, medical providers frequently turn to alternative therapies to treat advanced prostate cancer patients^43,44^. Cisplatin, a chemotherapy drug that halts tumor progression by leading to cancer cell apoptosis, is proved to have a moderate effect on metastatic castration-resistant prostate cancer^45^. Although cisplatin is not considered to use solely, it is confirmed that cisplatin has a significant synergistic effect with other chemotherapy drugs^46^.

It has been reported that miR-205 can enhance cisplatin toxicity in prostate cancer. MiR-205 can impair the autophagic pathway of prostate cancer by downregulating the lysosome-associated protein RAB27A/LAMP3 and eventually overcome the cisplatin resistance^47^. Conversely, another six oncogenic miRNAs, which derive from the miR-17–92 cluster, promote chemoresistance through activating the AKT pathway^48^ (Table 3).

## LncRNA and drug resistance

LncRNAs are a subset of non-coding RNAs whose length is more than 200nt. Although they share many similarities with other protein-coding mRNA, lncRNAs generally have limited or no protein-coding capacity^49^. LncRNAs can regulate processes such as chromatin remodeling, histone modifications, miRNAs sponging, mediation of complex formation and so on^50–53^. However, with the deeper study of lncRNA, it has gradually been revealed that it is vital in tumorigenesis, including in prostate cancer. Furthermore, lncRNAs play an important part in drug resistance of prostate cancer. Here, we present some lncRNAs that are crucial in drug resistance of prostate cancer (Table 4).

**Table 4:** LncRNA and drug resistance in prostate cancer.

LncRNAs	Expression	Genes and pathways	Drug	Reference
HOXD-AS1	Up	WDR5/H3K4me3	Bicalutamide	^60^
LncRNA-HOTAIR	Up	AR	Androgen	^62^
Lnc-LBCS	Down	hnRNPK/AR	Androgen	^107^
LncRNA-BCAR4	Up	GLI2	Androgen	^54^
LncRNA-SNHG6	Up	miR-186/CD51	Androgen	^55^
Linc00675	Up	MDM2/GATA2/AR	Androgen	^108^
Linc00518	Up	miR-216b-5p/GATA	Paclitaxel	^63^
LncRNA-CCAT1	Up	miR-24–3p/FSCN1	Paclitaxel	^64^
LncRNA-NEAT1	Up	miR-34a-5p and miR-204–5p/ASCL4	Docetaxel	^70^
LncRNA-MALAT1	Up	miR-145–5p/AKAP12	Docetaxel	^61,71^
LncRNA-DANCR	Up	miR-34a-5p/JAG1 or miR-135a	Docetaxel	^65^
LncRNA-CASC2	Down	miR-183/SPRY2	Docetaxel	^66^
LncRNA-HORAS5	Up	BCL2A1	Cabazitaxel	^109^
LncRNA-HOTTIP	Up	Wnt/β-catenin	Cisplatin	^72^
LOXL1-AS1	Down	miR-let-7a-5p/EGFR	Doxorubicin	^76^

### LncRNA and resistance to anti-androgen drugs

Several lncRNAs have been reported as highly expressed in CRPC. BCAR4, which has a crucial role in tamoxifen-resistance breast cancer, can bind to the promoter region of GLI2 and activate the GLI2 downstream genes, making the prostate cancer cells less sensitive to androgen stimulation^54^. Similarly, Lnc-SNHG17, which was identified as highly expressed in CRPC, can serve as a competing endogenous RNA (ceRNA) to sponge miR-144 in CRPC. Subsequently, inhibition of miR-144 upregulates the downstream target, CD51, to accelerate the CRPC cell proliferation and invasion^55^.

Apart from the above lncRNAs, there are several well-known lncRNAs have been reported to participate in the progression of CRPC. LncRNA HOXD-AS1, which has been extensively studied in colorectal carcinoma, glioma, cervical cancer, and liver cancer^56–59^, can promote castration by recruiting WDR5 to mediate histone 3 lysine 4 tri-methylation and then to regulate the downstream genes, such as PLK1, AURKA, and CDC25C^60^. Another famous lncRNA, MALAT1, is involved in CRPC progression both in vivo and in vitro. Silencing of MALAT1 can inhibit CRPC cell proliferation by arresting the CRPC cell in the G0/G1 cycle, Moreover, xenografts assays produced the same results^61^.

Meanwhile, LncRNA can directly bind to the androgen receptor to affect castration. Lnc-HOTAIR has been found to interact with the AR and prevent it from ubiquitination and degradation, and thus drives CRPC progression. Another LncRNA, Lnc-LBCS, can directly interact with hnRNPK and AR mRNA to form a complex and inhibit translation of the AR. Therefore, inhibiting the expression of LBCS can activate AR signaling to sustain the trait of castration^62^.

### LncRNA and resistance to taxane

Similarly, lncRNAs have a unique role in taxane resistance. Linc00518 was previously found to be highly expressed both in cancer cell lines and tumor tissues previously. Recent studies revealed that linc00518 can promote paclitaxel resistance through a sponge mechanism. Linc00518 regulates GATA6 expression and promotes paclitaxel resistance by competitively binding to miR-216b-5p^63^. Another lncRNA, CCAT1, also appears to enhance paclitaxel resistance in prostate cancer. Similarly, CCAT1 can sponge miR-24-3p and thus upregulate FSCN1 expression to promote paclitaxel resistance^64^.

Besides taking part in paclitaxel resistance, lncRNA also plays a vital role in prostate cancer resistance to docetaxel. LncRNA DANCR is significantly upregulated in docetaxel-resistant prostate cancer. Silencing of DANCR re-sensitized DTX-tolerant prostate cancer cells to docetaxel treatment. Further studies found that DANCR suppressed the miR-34a-5p-induced JAG1 degradation to trigger docetaxel resistance^65^. Another lncRNA, CASC2, also functions as a ceRNA for miR-183 to positively upregulate the expression of SPRY2, a key antagonist of RTK signaling, and enhance the cytotoxicity of docetaxel in prostate cancer^66^. Another famous lncRNA NEAT1, which is encoded from the familial tumor syndrome multiple endocrine neoplasia type 1 locus, is shown to exert oncogenic effects in many malignancies, including non-small-cell lung cancers, gastric cancers, and esophageal cancers^67–69^. A recent study showed that NEAT1 contributes to docetaxel resistance by upregulating ACSL expression; it sponges miR-34a-5p and miR-204-5p in prostate cancer^70^. Interestingly, lncRNA-MALAT1, which is proved to be involved in CRPC progress, enhances docetaxel resistance both in vivo and in vitro. Further research verified that MALAT1 upregulates AKAP12 expression via directly targeting miR-145-5p to promote DTX-chemoresistance^71^.

### LncRNA and resistance to cisplatin

It is reported that lncRNAs are also involved in cisplatin resistance. Lnc-HOTTIP is evidently highly expressed in prostate cancer samples compared to controls. Interestingly, HOTTIP also sustains prostate cancer cisplatin resistance through decreasing the apoptosis of PCa cells. Furthermore, the mRNA and protein levels of Cyclin D1, CDK4, and β‐catenin are reduced significantly by silencing the expression of HOTTIP, indicating that HOTTIP is involved in cisplatin resistance through regulating Wnt /β-catenin signaling^72^.

### LncRNA and resistance to other drugs

Both taxanes and anthracyclines have been well studied in CRPC. However, little attention has been focused on anthracyclines in prostate cancer compared to taxanes^73^. Doxorubicin is belonging to anthracyclines which is the first-line clinical drug for CRPC. Doxorubicin exerts its anti-tumor effect on a moleular level by blocking DNA replication and transcription to induce cancer cell apoptosis^74,75^. It is reported that lncRNA LOXL1-AS1 can promote doxorubicin resistance through the lncRNA LOXL1-AS1/miR-let-7a-5p/EGFR axis. And inhibition of lncRNA LOXL1-AS1 can be a potential strategy for drug-resistant prostate cancer patients^76^.

## CircRNA and drug resistance

Circular RNA is a subset of noncoding RNAs that is produced by a non-canonical splicing event called back-splicing. During back-splicing, a downstream 5’ splice site is joined to an upstream 3’ splice site to form circular RNAs^77,78^. Due to differences in their production, structure, and turnover, circular RNAs have many unique and important biological functions^79^. CircRNAs can function as decoys for miRNAs or proteins, act as scaffolds for circRNA-protein complex and recruit protein to specific loci^80–82^. In addition, some circRNAs can also be translated to produce small unique peptides^83,84^. In-depth research has gradually lead to a recognition that circRNAs play a vital role in chemoresistance in prostate cancer. We will list some representative circRNAs involved in drug resistance in prostate cancer below (Table 5).

**Table 5:** CircRNA and drug resistance in prostate cancer.

CircRNAs	Expression	Genes and pathways	Drug	Reference
hsa_circ_0004870	Down	RBM39	Enzalutamide	^86^
hsa_circ_0001427	Down	miR-181c-5p/AR-v7	Enzalutamide	^85^
hsa_circ_0000735	Up	miR-7	Docetaxel	^87^
circFoxo3	Down	Foxo3/EMT	Docetaxel	^88^

### CircRNA and resistance to anti-androgen drugs

Several circRNAs have been found to suppress enzalutamide-resistance prostate cancer cell progression. hsa_circ_0001427 have been found down-regulated in PCa specimens with higher Gleason scores, and the results from cell lines also confirmed that hsa_circ_0001427 is decreased in Enz-resistant CRPC cells compared to Enz-sensitive CRPC cells. Mechanism assays revealed that hsa_circ_0001427 can regulate AR-v7 expression by sponging miR-181c-5p. These results suggested that hsa_circ_0001427/miR-181c-5p/AR-v7 signaling could be a potential target for treatment of Enz-resistant PCa^85^. In addition, a circRNA microarray assay was performed to identify differentially expressed circRNAs between the Enz-resistant cell line and sensitive cell line. Hundreds of circRNAs, such as hsa_circ_0001721 and hsa_circ_0004870 were differentially expressed. Also, hsa_circ_0004870 was confirmed negatively correlated with AR and AR-v7 expression^86^.

### CircRNA and resistance to taxane

Circular RNA hsa_circ_0000735 is upregulated in docetaxel-resistant PCa tissues and cells. Functional assays have verified that silencing of hsa_circ_0000735 restrains DTX resistance and inhibits tumor progression. Moreover, hsa_circ_0000735 can serve as a sponge for miR-7, which is downregulated in DTX-resistant PCa, to promote PCa chemoresistance^87^. Another circRNA, circFOXO3, which is one of the most studied circRNAs, has been found to inhibit prostate cancer progression and docetaxel resistance through enhanced FOXO3 expression and repression of EMT^88^.

## Conclusions and future perspectives

**Fig. 2:**
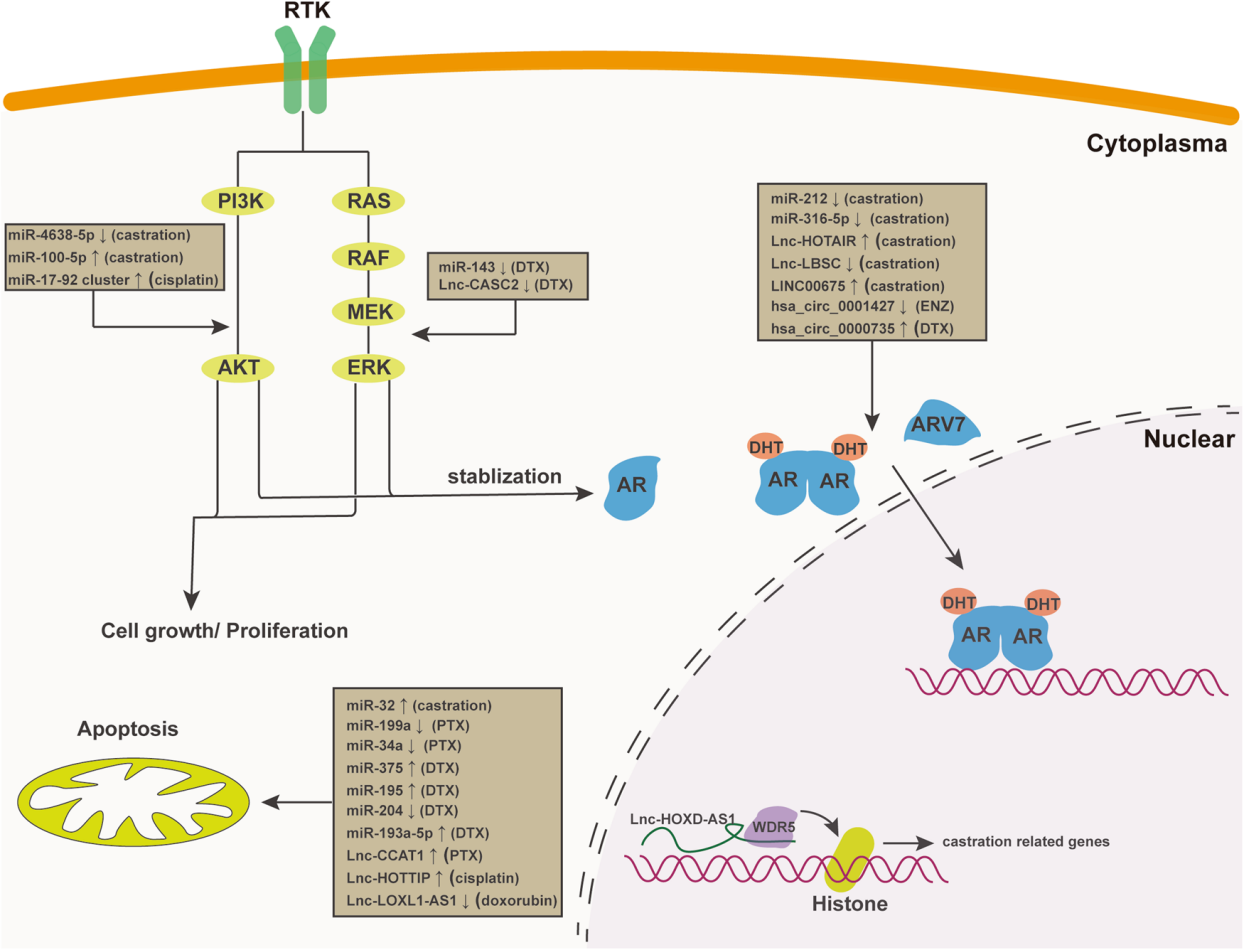
Schematic presentation of noncoding RNAs which participate in drug resistance of prostate cancer. A summary diagram of noncoding RNAs participated in the drug resistance of prostate cancer. Several miRNAs, lncRNAs and circRNAs have been found to participate in drug resistance by influencing RTK pathway related genes, apotptosis, AR-v7 and histone modification.

Since noncoding RNAs play significant roles in tumorigenesis, more and more attention has been focused on the relationship between noncoding RNA and chemoresistance. Accumulating evidences reveal that noncoding RNA (including miRNAs, lncRNAs, and circRNAs) has been involved in chemoresistance through targeting multiple signaling pathways (Fig. 2). Therefore, correcting the aberrant expression of noncoding RNA could be a promising strategy to overcome the chemoresistance of prostate cancer.

Compared with protein-coding genes, which have been extensively utilized as tumor therapy targets, noncoding RNA therapy has several advantages. Many proteins (80–85%) are “undruggable” due to lack of suitable structural features to interact with drug-like chemical compounds^89,90^. Noncoding RNAs, on the other hand, exist in nearly 98% of the genome. Therefore, noncoding RNAs could be more accessible targets for tumor therapy^91^. Also, nearly all traditional drugs for cancer therapy are facing drug resistance, whereas there are no drug resistance reports for noncoding RNA therapy so far. In addition, after chemical modification of noncoding RNA, the half-time of ncRNA drugs is longer than that of small molecules or antibodies^92,93^. Like antisense oligonucleotides (ASOs) with 2′-O-methoxyethyl modification in the backbone, can be more resistance to nucleases degradation^94,95^.

Despite the promising prospect for tumor therapy, there are still various obstacles for noncoding RNA therapy. Taking miRNAs as an example, though there are various ongoing clinical trials, no miRNAs have yet been applied in clinical use. There are still several difficulties to be overcome. First, it is of great concern to identify the best miRNA candidates for cancer therapy. Since noncoding RNAs normally targeting many sites, it is quite difficult to avoid an off-target effect in cells. Moreover, noncoding RNA may have the opposite effects in different tissues. As an example, miR-375 can promote docetaxel resistance in prostate cancer, as mentioned above. However, miR-375 has been reported to facilitate osteosarcoma progression^96^, suggesting that it plays a different role in different systems. Thus, ncRNA therapy should comprehensively consider the overall effect of agents in the human body. Secondly, a more efficient delivery system is also required to maintain the therapeutic oligonucleotides treatment efficiency and decrease toxicity to other organs. Even though great efforts have been made to design distinct RNA oligos or different polymer coatings to treat tumors more specifically, viral agents (for example, CRISPR–Cas systems) or nanoparticles agents are still the most commonly employed delivery methods for noncoding RNA therapy^97^. Thus, toxicity and immune responses are still difficult to overcome. In summary, there is still a long way to go before noncoding RNA therapy can be applied in practice. As for lncRNAs and circRNA, more researches should be conducted to better understand the underlying mechanism in chemoresistance of prostate cancer.

Currently, there are several ongoing ncRNA therapy clinical trials using locked nucleic acid (LNA) technology to manipulate the expression of noncoding RNAs. One of the most famous miRNA formulations, MRX34 (a liposomal of miR-34a mimic) has undergone phase I clinical trials in the U.S and Korea with advanced hepatoma, melanoma, renal cell carcinoma, and other cancers. Although 16 of 46 patients remained in stable conditions for at least 4 weeks, these clinical trials were ultimately terminated due to several adverse immune-related effects. However, another LNA-technology-based drug, which targets miR-122 in hepatitis C infection patients, has been proved to effectively reduce the HCV RNA in phase II trials without unmanageable adverse events. These active ncRNA-related clinical trials show that noncoding RNA has good prospects for treatment of cancer, infection, and other “undruggable” diseases. Ultimately, the off-target toxicity and immune response are still the ongoing challenges for clinical treatment.

Looking forward, with the development of noncoding RNA delivery systems and fewer off-target effects, we imagine that whole-genome sequencing (including noncoding RNA) may be necessary for individual cancer therapy in the near future. We also believe that noncoding RNA therapy could be an effective supplement for traditional treatments.
